# Inverse problems of combined photoacoustic and optical coherence tomography

**DOI:** 10.1002/mma.3915

**Published:** 2016-03-17

**Authors:** Peter Elbau, Leonidas Mindrinos, Otmar Scherzer

**Affiliations:** ^1^ Computational Science CenterUniversity of ViennaOskar‐Morgenstern‐Platz 1 ViennaA‐1090Austria; ^2^ Johann Radon Institute for Computational and Applied Mathematics (RICAM)Altenbergerstraße 69LinzA‐4040Austria

**Keywords:** quantitative photoacoustic tomography, optical coherence tomography, hybrid imaging

## Abstract

Optical coherence tomography (OCT) and photoacoustic tomography are emerging non‐invasive biological and medical imaging techniques. It is a recent trend in experimental science to design experiments that perform photoacoustic tomography and OCT imaging at once. In this paper, we present a mathematical model describing the dual experiment. Because OCT is mathematically modelled by Maxwell's equations or some simplifications of it, whereas the light propagation in quantitative photoacoustics is modelled by (simplifications of) the radiative transfer equation, the first step in the derivation of a mathematical model of the dual experiment is to obtain a unified mathematical description, which in our case are Maxwell's equations. As a by‐product, we therefore derive a new mathematical model of photoacoustic tomography based on Maxwell's equations. It is well known by now that without additional assumptions on the medium, it is not possible to uniquely reconstruct all optical parameters from either one of these modalities alone. We show that in the combined approach, one has additional information, compared with a single modality, and the inverse problem of reconstruction of the optical parameters becomes feasible. © 2016 The Authors. *Mathematical Methods in the Applied Sciences* Published by John Wiley & Sons Ltd.

## 1 Introduction

Only recently, there have been developed experimental setups that can perform photoacoustic and optical coherence tomography experiments in parallel; see 1 and further developments in 2, 3. Combined setups can be used for imaging biological tissue up to a depth of approximately 5 mm. They have been used for *in vivo* studies of human and mouse skins. In the current state of experiments, the two recorded modalities are visualised by superposition after registration, and we refer the reader to the review paper 4.

In this paper, we derive a mathematical model for quantitative imaging of the multi‐modal experiment based on Maxwell's equations. This study characterises the additional information obtainable by the dual experiment. In this paper, we assume that the sample is an inhomogeneous, isotropic, non‐magnetic and linear dielectric medium, and the imaging parameters of the medium are the conductivity, the susceptibility and the Grüneisen parameter.

Even though the mathematical modelling of optical coherence tomography based on Maxwell's equations is well established, most of the literature relies on simplified models of the Helmholtz equation 5, 6, 7, 8, 9, where, as a consequence, the frequency dependence of the susceptibility is neglected. It is a recent trend to consider the more general case of Maxwell's equations for the mathematical description of the optical coherence tomography (OCT) system 10, 11, 12, 13.

The mathematical modelling of quantitative photoacoustics, on the other hand, is based on the radiative transfer equation or its diffusion approximation 14, 15. Maxwell's equations have only been considered for mathematical models in thermoacoustic tomography where low‐frequency radiation is used for illumination 16, 17.

To formulate the dual‐modal setup, we consider Maxwell's equations as the basic modelling equations because both imaging techniques rely on the same excitation. In particular, this provides a more general model for quantitative photoacoustics based on Maxwell's equations, which is also applicable in the case of high frequency radiation.

This paper is organised as follows. In Section 2, we give an overview of the inverse problem in photoacoustics. We present the governing equations and the commonly used simplifications. Next, we formulate mathematically the problem of optical coherence tomography starting from Maxwell's microscopic equations. For an extensive mathematical modelling of optical coherence tomography, we refer to 11. The system of equations for the dual experiment is obtained in Section 4. In Section 5, the equation system is transformed to a Fredholm integral equation for the Grüneisen parameter under the far field and Born approximations, respectively. Finally, we discuss the connection between the optical parameters appearing in the radiative transfer equation and Maxwell's equations. To simplify the reading, we summarise the basic notations used in this paper in Table 1.

**Table 1:** Physical quantities.

Symbol	Quantity	Definition
*c*	speed of light	(1)
*μ*_a_	absorption coefficient	(1)
*μ*_s_	scattering coefficient	(1)
*h*	Planck constant	(2)
*ε*	diffusion coefficient	(4)
*γ*	Grüneisen parameter	(6)
*K*	Bulk modulus	(7)
*ϱ*	mass density	(7)
*c*_*s*_	speed of sound	(8)
*μ*	electric permittivity	(20)
*σ*	electric conductivity	(21)
*χ*	electric susceptibility	(21)

## 2 Photoacoustic imaging

Photoacoustic tomography is a hybrid imaging technique, which measures the acoustic response of an object upon illumination with an electromagnetic wave. In the experiments, the object is illuminated with a short laser pulse, typically with light in the near infrared spectrum. This pulse is absorbed by the medium, where the exact amount varies locally depending on the material properties, in particular on the absorption coefficient, and thereby, a fraction is transformed into heat energy. This leads to a local change of pressure in the medium, where the transformation factor is again depending on the local thermodynamic properties of the medium, typically reduced to the single Grüneisen parameter. Finally, this pressure propagates as an ultrasonic wave through the medium and is recorded at every point on a surface around the object as a function of time.

For a mathematical description of this measurement setup, we have to model the three different phenomena:

- the propagation of the laser light in the medium;
- the transformation of the absorbed energy into a pressure distribution; and
- the propagation of the pressure wave inside the medium.

For the reader's convenience, we summarise in Table 2 the variables, which are used throughout this section, as a backup reference.

**Table 2:** Radiative transfer equation.

Symbol	Quantity	Definition
*ψ*_***ϑ***_	photon density	(1)
Θ	phase function	(1)
Φ_***ϑ***_	energy fluence	(2)
$\bar{\Phi }$	total energy fluence	(5)

The light propagation is usually modelled via the radiative transfer equation. Assuming that we have a laser pulse of fixed frequency *ν* > 0, the photon density *ψ*_***ϑ***_(*t*,***x***) at time t∈R and coordinate $x\in R^{3}$ moving in the direction $\vartheta \in S^{2}$ fulfils the equation

(1) $$\frac{1}{c}\partial_{t}\psi_{\vartheta }(t,x)+<\vartheta ,\nabla_{x}\psi_{\vartheta }(t,x)>+(\mu_{s}(x)+\mu_{a}(x))\psi_{\vartheta }(t,x)=\frac{\mu_{s}(x)}{4\pi }\underset{S^{2}}{\int }\Theta (x,\tilde{\vartheta },\vartheta )\psi_{\tilde{\vartheta }}(t,x)\;ds(\tilde{\vartheta }),$$

where *μ*_a_ is the absorption coefficient and *μ*_s_ is the scattering coefficient at the radiation frequency *ν*; see for example 18. Here, the constant *c* denotes the speed of light, and Θ is the phase function, that is, $\Theta (x,\tilde{\vartheta },\vartheta )$ is the probability density that a photon heading into a direction $\tilde{\vartheta }\in S^{2}$ is scattered at the position $x\in R^{3}$ into the direction $\vartheta \in S^{2}$.

Because in photoacoustic imaging, the laser excitation occurs to be very short and the absorption of the light happens much faster than the propagation of the acoustic wave, the light distribution as a function of time is not relevant, but only the total energy absorbed at each point matters. Thus, we turn our attention to the total energy fluence Φ_***ϑ***_ originating from photons moving in the direction $\vartheta \in S^{2}$ as new variable:

(2) $$\Phi_{\vartheta }(x)=\mathrm{h\nu}\overset{\infty }{\underset{-\infty }{\int }}\psi_{\vartheta }(t,x)c\;dt,\;x\in R^{3},\;\vartheta \in S^{2},$$

where *h* denotes the Planck constant. Then, Φ_***ϑ***_ solves the equation

(3) $$<\vartheta ,\nabla_{x}\Phi_{\vartheta }(t,x)>+(\mu_{s}(x)+\mu_{a}(x))\Phi_{\vartheta }(t,x)=\frac{\mu_{s}(x)}{4\pi }\underset{S^{2}}{\int }\Theta (x,\tilde{\vartheta },\vartheta )\Phi_{\tilde{\vartheta }}(t,x)\;ds(\tilde{\vartheta }).$$

However, because the phase function Θ is unknown, often the diffusion approximation

$$\text{div}(\varepsilon \nabla \bar{\Phi })(x)=\mu_{a}(x)\bar{\Phi }(x),$$

with the diffusion coefficient *ε* given by

(4) $$\epsilon (x)=[3{\left(\mu_{\text{a}}(x)+\left(1-\frac{1}{3}\Theta_{1}(x)\right)\mu_{\text{s}}(x)\right]}^{-1}$$

is used, which is an equation for the total energy fluence

(5) $$\bar{\Phi }(x)=\frac{1}{4\pi }\underset{S^{2}}{\int }\Phi_{\vartheta }(x)\;ds(\vartheta ).$$

This is justified if $\Theta (x,\tilde{\vartheta },\vartheta )\approx 1+\Theta_{1}(x)〈\tilde{\vartheta },\vartheta 〉$ and $\Phi_{\vartheta }(x)\approx \bar{\Phi }(x)+<\phi_{1}(x),\vartheta >$ for some functions Θ_1_ and ***ϕ***_1_, which is a good approximation if we have a strongly scattering medium; see 19, Chapter XXI, §5].

The absorbed energy at a point ***x*** is then given by $\mu_{a}(x)\bar{\Phi }(x)$. Moreover, it is common to assume that the produced pressure density *p* ^(0)^ inside the medium is proportional to the absorbed energy where the proportionality coefficient is the Grüneisen parameter *γ*:

(6) $$p^{\left(0\right)}(x)=\gamma (x)\mu_{a}(x)\bar{\Phi }(x).$$

For the propagation of the acoustic wave, the standard assumption is that we have an elastic medium with mass density *ϱ*, bulk modulus *K* and vanishing shear modulus. Then, according to linear elasticity theory, for instance 20, we obtain the equation

(7) $$\partial_{\mathrm{tt}}p(t,x)=K(x){\text{div}}_{x}\left(\frac{1}{\varrho }\nabla_{x}p\right)(t,x).$$

Moreover, the density is usually considered to be approximatively constant throughout the medium, so that we may simplify this equation to the linear wave equation

(8) $$\partial_{\mathrm{tt}}p(t,x)=c_{s}^{2}(x)\Delta_{x}p(t,x)$$

with the speed of sound $c_{s}=\sqrt{\frac{K}{\varrho }}$.

Finally, we acquire the measurements

(9) g(t,ξ)=p(t,ξ),t>0,ξ∈D

at some detector surface $D\subset R^{3}$.

Putting all this together, the inverse problem of photoacoustic imaging is to recover the material parameters *μ*_a_, *μ*_s_ (which in this approximation only enters via the diffusion coefficient *ε*; see (4)), *γ* and *c*_s_ from the given measurement data *g*.

This problem can be solved in two steps. First, we consider the acoustic part, given by the equation system

(10) $$\begin{array}{ccc} \partial_{\mathrm{tt}}p(t,x) & =c_{s}^{2}(x)\Delta_{x}p(t,x),\; & t>0,\;x\in R^{3},\\ \partial_{t}p(0,x) & =0,\; & x\in R^{3},\\ p(t,\xi ) & =g(t,\xi ),\; & t>0,\;\xi \in D,\\ p(0,x) & =p^{\left(0\right)}(x),\; & x\in R^{3}. \end{array}$$

For given, non‐trapping speed of sound *c*_s_ (in practice, a common assumption is that *c*_s_ is approximatively constant), the measurements *g* allow to uniquely reconstruct the initial pressure *p* ^(0)^; see 21. If the detector surface has a simple geometry (the easiest examples are a planar or a spherical detector) and the speed of sound is assumed to be constant, then there are a lot of explicit reconstruction formulas for *p* ^(0)^ available: 22, 23, 24, 25, 26, 27, 28, 29, 30, 31, 32, 33.

For unknown speed of sound, it was suggested in 34 to use multiple photoacoustic measurements with a focussed illumination; see also 35, 36 for reconstruction formulas for this kind of illumination. For a more extensive review of these works, we refer to 37.

So, let us assume that we can invert the acoustic problem and recover *p* ^(0)^ from the photoacoustic measurements. Then, as the second step, it remains the problem of reconstructing the material parameters *μ*_a_, *μ*_s_, *γ* from the internal data *p* ^(0)^ via the relations

$$\begin{array}{ccc} {\text{div}}_{x}(\varepsilon \nabla \bar{\Phi })(x) & =\mu_{a}(x)\bar{\Phi }(x),\; & x\in R^{3},\\ \bar{\Phi }(\xi ) & ={\bar{\Phi }}^{\left(0\right)}(\xi ),\; & \xi \in D,\\ p^{\left(0\right)}(x) & =\gamma (x)\mu_{a}(x)\bar{\Phi }(x),\; & x\in R^{3}, \end{array}$$

where we assumed that we know the boundary data ${\bar{\Phi }}^{\left(0\right)}$ at the detector surface, because we are expected to know all the material parameters outside the object, in particular in the vicinity of the detector, and thus, *p* ^(0)^ allows us to calculate $\bar{\Phi }$ around the detector surface. However, in practice, these data are not used, because the absorption is considered very small outside the object of interest. We are only using these data mathematically to be able to define the modelling equations in all $R^{3}$, which is state of the art in this field.

In 38, it was shown that even with multiple photoacoustic measurements, it is not possible to reconstruct all three parameters, but from the knowledge of one of the parameters (usually, the Grüneisen parameter *γ* is assumed to be approximatively constant, although there is no physical evidence for this), the other two can be explicitly calculated; see also 39 for a review about the reconstruction from internal data.

Thus, the inverse photoacoustic problem is not well posed, as it requires an additional three‐dimensional data to allow the reconstruction of all material parameters.

## 3 Optical coherence tomography

Opposed to photoacoustic imaging, optical coherence tomography is not a hybrid imaging technique. It visualises the back‐scattered light of an object. However, analogously to photoacoustic imaging, the object is illuminated with a short laser pulse in the visible or near infrared spectrum. In practical realisations, the illumination is triggered to produce a plane wave, and the scattered wave is measured at a planar detector parallel to the plane wave illumination.

Because the time resolution of the detectors, which would be required for the desired spatial resolution, is practically hard to achieve, the detection of the reflected wave is accomplished by superimposing it with a reference beam, which is a copy of the wave used for illumination (produced by a beam splitter from the original wave) sent to a mirror instead of to the object. Then, the total intensity of this superposition is measured at every point on the detection plane.

For the reader's convenience, we summarise the variables used in this section, in Table 3.

**Table 3:** Electromagnetic waves.

Symbol	Quantity	Definition
***e***	microscopic electric field	(11a)
*ρ*	microscopic charge density	(11a)
***b***	microscopic magnetic field	(11b)
***j***	microscopic electric current	(11d)
***E***	averaged electric field	(15a)
$\bar{\rho }$	averaged charge density	(15a)
***B***	averaged magnetic field	(15b)
$\bar{j}$	averaged electric current	(15d)
$\tilde{\rho }$	approximation of $\bar{\rho }$	(16a)
***J***	approximation of $\bar{j}$	(16b)
***P***	electric polarisation	(18)
***M***	magnetic polarisation	(19)
***E***_*s*_	stationary electric field	(22)
***B***_*s*_	stationary magnetic field	(22)
***j***_*s*_	stationary electric current	(22)
*ρ*_*s*_	stationary charge density	(22)
E	electrostatic potential energy	(42)
W	total electromagnetic energy density	(48)
***S***	Poynting vector	(48)
***D***	electric displacement	(49)

### 3.1 Microscopic Maxwell's equations

The interaction of the laser pulse, which is an electromagnetic wave, and the medium is best described by Maxwell's microscopic equations. These are equations for the time evolution of the microscopic electric and magnetic fields ***e*** and ***b*** for given microscopic charge density *ρ* and microscopic electric current ***j***:

(11a) $${\text{div}}_{x}e(t,x)=4\pi \rho (t,x),\;\;\;\;t\in R,\;x\in R^{3},$$

(11b) $${\text{div}}_{x}b(t,x)=0,\;\;\;\;\;\;\;t\in R,\;x\in R^{3},$$

(11c) $${\text{curl}}_{x}e(t,x)=-\frac{1}{c}\partial_{t}b(t,x),\;\;\;\;\;t\in R,\;x\in R^{3},$$

(11d) $${\text{curl}}_{x}b(t,x)=\frac{1}{c}\partial_{t}e(t,x)+\frac{4\pi }{c}j(t,x),\;t\in R,\;x\in R^{3}.$$

Remark 3.1 If the initial data ***e***(0,·) and ***b***(0,·) are compatible (that is, they are satisfying the Eqns (11a) and (11b) at *t* = 0) and the continuity equation

(12) $$\partial_{t}\rho (t,x)+{\text{div}}_{x}j(t,x)=0,\;t\in R,\;x\in R^{3},$$

holds, then the Eqns (11c) and (11d) imply (11a) and (11b).

To see this, take the divergence of the two vector Eqns (11c) and (11d). Then, using the identity div_***x***_ **curl**_***x***_ ***f*** = 0 for every vector field ***f***, it follows with (12) that

$${\text{div}}_{x}\partial_{t}b=0\;\text{and}\;{\text{div}}_{x}\partial_{t}e=-4\pi {\text{div}}_{x}j=4\pi \partial_{t}\rho .$$

Integrating these equations over time gives the Eqns (11a) and (11b).

To obtain a formula for the evolution of the charge density *ρ* and the electric current ***j***, we consider a medium that consists of

- *N* charged particles,
- at the positions $x_{i}:R\to R^{3}$, *i* = 1,…,*N*, as functions of time,
- with masses *m*_*i*_ and
- charge densities *q*_*i*_ *ρ*_*i*_, where $q_{i}\in R$ and $\rho_{i}\in C_{c}^{\infty }(R^{3})$ are non‐negative functions with $∥\rho_{i}{∥}_{L^{1}}=1$. Because these particles have physical radii smaller than 10^−14^m, *ρ*_*i*_ can be considered an approximation of a *δ*‐distribution.

Then, the charge density *ρ* and the electric current ***j*** are given by the following:

(13a) $$\rho (t,x)=\overset{N}{\underset{i=1}{\sum }}q_{i}\rho_{i}(x-x_{i}(t)),\;\text{and}$$

(13b) $$j(t,x)=\overset{N}{\underset{i=1}{\sum }}q_{i}x_{i}^{\prime }(t)\rho_{i}(x-x_{i}(t)).$$

Clearly, they fulfil the continuity Eqn (12).

For the motion of the particles, we assume that they move according to the Lorentz force:

(14) $$m_{i}x_{i}^{\prime \prime }(t)=q_{i}\underset{R^{3}}{\int }e(t,x)\rho_{i}(x-x_{i}(t))\;dx+q_{i}\frac{x_{i}^{\prime }(t)}{c}\times \underset{R^{3}}{\int }b(t,x)\rho_{i}(x-x_{i}(t))\;dx.$$

The Eqns (11), (13) and (14) completely define the evolution of the medium starting from initial conditions ***x***_*i*_(0) and $x_{i}^{\prime }(0)$, *i* = 1,…,*N* for the particles and ***e***(0,***x***) and ***b***(0,***x***), $x\in R^{3}$, for the microscopic electromagnetic fields; see for example 40, Chapter 6].

### 3.2 Macroscopic Maxwell's equations

However, solving the huge system of Eqns (14) is practically impossible. Therefore, we average the fields with respect to the space variable by some non‐negative weighting function $w\in C_{c}^{\infty }(R^{3})$ with support around 0 and $∥w{∥}_{L^{1}}=1$:

$$\begin{array}{cccc} E(t,x) & =\underset{R^{3}}{\int }w(y-x)e(t,y)\;dy, & B(t,x) & =\underset{R^{3}}{\int }w(y-x)b(t,y)\;dy,\\ \bar{j}(t,x) & =\underset{R^{3}}{\int }w(y-x)j(t,y)\;dy, & \bar{\rho }(t,x) & =\underset{R^{3}}{\int }w(y-x)\rho (t,y)\;dy. \end{array}$$

Then, taking the average of the equation system (11) with respect to the function *w*, we obtain Maxwell's macroscopic equations

(15a) $${\text{div}}_{x}E(t,x)=4\pi \bar{\rho }(t,x),\;\;\;\;t\in R,\;x\in R^{3},$$

(15b) $${\text{div}}_{x}B(t,x)=0,\;\;\;\;\;\;\;t\in R,\;x\in R^{3},$$

(15c) $${\text{curl}}_{x}E(t,x)=-\frac{1}{c}\partial_{t}B(t,x),\;\;\;\;\;\;\;t\in R,\;x\in R^{3},$$

(15d) $${\text{curl}}_{x}B(t,x)=\frac{1}{c}\partial_{t}E(t,x)+\frac{4\pi }{c}\bar{j}(t,x),\;\;\;\;t\in R,\;x\in R^{3},$$

for the averaged electromagnetic fields ***E*** and ***B*** depending on the averaged charge density $\bar{\rho }$ and the averaged electric current $\bar{j}$.

Remark 3.2 Because the medium usually consists of molecules, most of the particles are clustered around the centres of these molecules. Let us relabel the particles ${\left(x_{i}\right)}_{i=1}^{N}$ therefore as ${\left({\left(x_{i,k}\right)}_{i=1}^{I_{k}}\right)}_{k=1}^{K}$ such that ***x***_*i*,*k*_ is the *i*th particle of the *k*th cluster of particles, *i* = 1,…,*I*_*k*_, *k* = 1,…,*K*, write *q*_*i*,*k*_ for its electric charge and let ${\bar{x}}_{k}$ be the mass centre of the *k*th molecule.

Then, we introduce the zeroth‐order approximations $\tilde{\rho }$ and ***J*** of the charge density $\bar{\rho }$ and the electric current $\bar{j}$, respectively, which are obtained from the averaged fields $\bar{\rho }$ and $\bar{j}$ when the molecules are assumed to be point like, that is, setting $x_{i,k}={\bar{x}}_{k}$ for all *i*:

(16a) $$\tilde{\rho }(t,x)=\overset{K}{\underset{k=1}{\sum }}w({\bar{x}}_{k}(t)-x)\overset{I_{k}}{\underset{i=1}{\sum }}q_{i,k},$$

(16b) $$J(t,x)=\overset{K}{\underset{k=1}{\sum }}{\bar{x}}_{k}^{\prime }(t)w({\bar{x}}_{k}(t)-x)\overset{I_{k}}{\underset{i=1}{\sum }}q_{i,k}.$$

We note that $\tilde{\rho }$ and ***J*** also fulfil the continuity equation

(17) $$\partial_{t}\tilde{\rho }(t,x)+{\text{div}}_{x}J(t,x)=0.$$

We now write the deviation $\bar{\rho }-\tilde{\rho }$ of the averaged charge density from its zeroth‐order approximation $\tilde{\rho }$ as the divergence of some function $P:R\times R^{3}\to R^{3}$, the so‐called electric polarisation:

(18) $$\bar{\rho }(t,x)=\tilde{\rho }(t,x)-{\text{div}}_{x}P(t,x).$$

According to Helmholtz's theorem, this is always possible but defines ***P*** only up to the addition of a divergence‐free vector field.

Then, we realise that we have with the continuity Eqns (12) and (17) and the definition (18)

$${\text{div}}_{x}\left(\bar{j}-J-\partial_{t}P\right)=-\partial_{t}(\bar{\rho }-\tilde{\rho }+{\text{div}}_{x}P)=0.$$

Doing the Helmholtz decomposition of the vector field $\bar{j}-J-\partial_{t}P$, we therefore find a function $M:R\times R^{3}\to R^{3}$, the magnetic polarisation, (uniquely defined up to a gradient vector field) so that

(19) $$\bar{j}(t,x)=J(t,x)+\partial_{t}P(t,x)+c\;{\text{curl}}_{x}M(t,x).$$

These decompositions (18) and (19) of $\bar{\rho }$ and $\bar{j}$ into macroscopic ( $\tilde{\rho }$ and ***J***) and polarisation (***P*** and ***M***) parts are standard in the physics literature to formulate Maxwell's macroscopic equations; see for example 40, Chapter 6.6].

Clearly, the equation system (15) is not enough to determine the averaged fields ***E*** and ***B***, because we would still require a solution of the microscopic problem to obtain the averaged quantities $\bar{\rho }$ and $\bar{j}$. The common way around this is to impose heuristic relations between these functions and the fields ***E*** and ***B***. A common choice for biological tissue is a linear relationship.

Definition 3.3 We call a material where

(20) $$\bar{j}(t,x)=\overset{\infty }{\underset{-\infty }{\int }}\mu (\tau ,x)E(t-\tau ,x)\;d\tau$$

for some function $\mu \in C_{c}^{\infty }(R\times R^{3})$ with *μ*(*τ*,***x***) = 0 for all *τ* < 0, $x\in R^{3}$ a non‐magnetic, isotropic and linear dielectric medium.

For a non‐magnetic, isotropic and linear dielectric medium, we may solve the equation system (15) for given parameter *μ* for ***E*** and ***B***.

Remark 3.4 In the standard notation introduced in Remark 3.2, in particular (18) and (19), the assumptions corresponding to (20) are such that no magnetic polarisation exists in the medium, that is, ***M*** = 0, and that the macroscopic current ***J*** and the electric polarisation ***P*** are of the form

(21) $$\begin{array}{cc} J(t,x) & =\overset{\infty }{\underset{-\infty }{\int }}\sigma (\tau ,x)E(t-\tau ,x)\;d\tau ,\\ P(t,x) & =\overset{\infty }{\underset{-\infty }{\int }}\chi (\tau ,x)E(t-\tau ,x)\;d\tau , \end{array}$$

where $\sigma \in C_{c}^{\infty }(R\times R^{3})$ is called the conductivity and $\chi \in C_{c}^{\infty }(R\times R^{3})$ is the electric susceptibility.

By (19), the relation between the function *μ*, defined in (20), and the pair *σ* and *χ* is simply

$$\mu (t,x)=\sigma (t,x)+\partial_{t}\chi (t,x).$$

### 3.3 Problem formulation

Initially, the electromagnetic fields should describe a laser pulse, which did not yet interact with the medium. Thus, we want to look for a solution (***E***,***B***), which for *t* < 0 is the superposition of a solution (***E*** ^(0)^,***B*** ^(0)^) of the vacuum equations (that is a solution of (15) with $\bar{\rho }=0$ and $\bar{j}=0$) with support outside the medium (that is outside the support of *μ*(*τ*,·) for every τ∈R) and a stationary solution (***E***_s_,***B***_s_) produced by the undisturbed medium (we want to assume that without exterior influence, the electric and magnetic fields generated by the medium do not vary in time).

Proposition 3.5 Let $\Omega \subset R^{3}$ be an open set. For given functions $\rho_{s}\in C_{c}^{\infty }(R^{3})$ and $j_{s}\in C_{c}^{\infty }(R^{3};R^{3})$ with supp *ρ*_s_⊂Ω, supp ***j***_s_⊂Ω, and div ***j***_s_=0, we define the stationary electromagnetic fields $E_{s},B_{s}\in C^{\infty }(R^{3};R^{3})$ by the following:

(22) $$\begin{array}{cc} \text{div}E_{s}(x) & =4\pi \rho_{s}(x),\;\text{div}B_{s}(x)=0,\\ \text{curl}\;E_{s}(x) & =0,\;\;\;\text{curl}\;B_{s}(x)=\frac{4\pi }{c}j_{s}(x). \end{array}$$

Moreover, let ***E*** ^(0)^ be a solution of the wave equation

(23) $$\frac{1}{c^{2}}\partial_{\mathrm{tt}}E^{\left(0\right)}(t,x)=\Delta_{x}E^{\left(0\right)}(t,x),\;t\in R,\;x\in R^{3},$$

with div_*x*_ ***E*** ^(0)^=0, $E^{\left(0\right)}(\cdot ,x)\in L^{1}(R)$ for every $x\in R^{3}$, and fulfilling that

(24) $$\text{supp}E^{\left(0\right)}(t,\cdot )\cap \Omega =\emptyset \text{for every}t\leq 0.$$

Finally, let $\mu \in C_{c}^{\infty }(R\times R^{3})$ fulfil supp *μ*(*t*,·)⊂Ω for all t∈R, *μ*(*t*,***x***) = 0 for all *t*≤0, $x\in R^{3}$, and

(25) $$j_{s}(x)=E_{s}(x)\overset{\infty }{\underset{0}{\int }}\mu (t,x)\;dt\;\text{for all}\;x\in R^{3}.$$

Then, there exists a solution ***E***, ***B*** of Maxwell's macroscopic equations (15) for a non‐magnetic, isotropic and linear dielectric medium with the parameter *μ*, as in Definition 3.3, and with charge density $\bar{\rho }(t,x)=\rho_{s}(x)-\overset{t}{\underset{-\infty }{\int }}{\text{div}}_{x}\bar{j}(\tau ,x)\;d\tau$ such that we have for all *t*≤0

(26) $$E(t,x)=E^{\left(0\right)}(t,x)+E_{s}(x)$$

(27) $$B(t,x)=-c\overset{t}{\underset{-\infty }{\int }}{\text{curl}}_{x}E^{\left(0\right)}(\tau ,x)\;d\tau +B_{s}(x).$$

In particular, ***E*** is the solution of the differential equation

(28) $$\frac{1}{c^{2}}\partial_{\mathrm{tt}}E(t,x)+{\text{curl}}_{x}\;{\text{curl}}_{x}E(t,x)=-\frac{4\pi }{c^{2}}\overset{\infty }{\underset{-\infty }{\int }}\partial_{t}\mu (\tau ,x)E(t-\tau ,x)\;d\tau$$

together with the condition (26), which is equivalent to the integral equation

(29) $$E(t,x)=E^{\left(0\right)}(t,x)+E_{s}(x)-\underset{R^{3}}{\int }\overset{t-\frac{\left|y\right|}{c}}{\underset{-\infty }{\int }}\frac{1}{\left|y\right|}\left({\mathrm{grad}}_{x}{\text{div}}_{x}-\frac{1}{c^{2}}\partial_{\mathrm{tt}}\right)\bar{j}(\tau ,x+y)\;d\tau \;dy,$$

where $\bar{j}$ is given by (20).

We first check that (26) and (27) solve Maxwell's equations (15) for *t*≤0. We have directly by construction

$${\text{div}}_{x}E(t,x)=4\pi \rho_{s}(x)\;\text{and}\;{\text{div}}_{x}B(t,x)=0,$$

which are the Eqns (15a) and (15b). Moreover, we have (15c), because

$$\partial_{t}B(t,x)=-c\;{\text{curl}}_{x}E^{\left(0\right)}(t,x)=-c\;{\text{curl}}_{x}E(t,x).$$

Finally, we obtain

$$\frac{1}{c}\partial_{t}E(t,x)=\frac{1}{c}\overset{t}{\underset{-\infty }{\int }}\partial_{\mathrm{tt}}E^{\left(0\right)}(\tau ,x)\;d\tau =c\overset{t}{\underset{-\infty }{\int }}\Delta_{x}E^{\left(0\right)}(\tau ,x)\;d\tau .$$

Because div_***x***_ ***E*** ^(0)^=0, we obtain with the identity **curl**_***x***_ **curl**_***x***_ ***F*** = **g** **r** **a** **d**_***x***_div_***x***_ ***F***−Δ_***x***_ ***F*** for every function $F\in C^{2}(R^{3};R^{3})$ that

$$\begin{array}{cc} \frac{1}{c}\partial_{t}E(t,x) & =-c\overset{t}{\underset{-\infty }{\int }}{\text{curl}}_{x}\;{\text{curl}}_{x}E^{\left(0\right)}(\tau ,x)\;d\tau \\ ={\text{curl}}_{x}B(t,x)-{\text{curl}}_{x}B_{s}(x)={\text{curl}}_{x}B(t,x)-\frac{4\pi }{c}j_{s}(x). \end{array}$$

It remains to check that the current $\bar{j}$, defined by relation (20), indeed fulfils $\bar{j}(t,x)=j_{s}(x)$ for *t*≤0. Using that *μ*(*t*,***x***) = 0 for *t*≤0, condition (24) and (25), we find

$$\begin{array}{cc} \overset{\infty }{\underset{-\infty }{\int }}\mu (t-\tau ,x)E(\tau ,x)\;d\tau & =\overset{0}{\underset{-\infty }{\int }}\mu (t-\tau ,x)(E^{\left(0\right)}(t,x)+E_{s}(x))\;d\tau \\ =E_{s}(x)\overset{0}{\underset{-\infty }{\int }}\mu (t-\tau ,x)\;d\tau =j_{s}(x). \end{array}$$

Thus, we have shown that (26) and (27) solve Maxwell's equations (15) for *t*≤0.

Combining Maxwell's macroscopic equations (15c) and (15d), it follows that ***E*** in particular solves the equation

$$\frac{1}{c^{2}}\partial_{\mathrm{tt}}E(t,x)+{\text{curl}}_{x}\;{\text{curl}}_{x}E(t,x)=-\frac{4\pi }{c^{2}}\partial_{t}\bar{j}(t,x)\text{for all}t\in R,x\in R^{3}.$$

To derive the integral equation, we use that ***E*** − ***E***_s_, ***B*** − ***B***_s_ is a solution of (15) with $\bar{\rho }$ replaced by $\bar{\rho }-\rho_{s}$ and $\bar{j}$ by $\bar{j}-j_{s}$. Therefore, according to the representation (A1) of the solution derived in Proposition A.1, we find for ***E*** that

$$\begin{array}{cc} E(t,x) & =E_{s}(x)+E^{\left(0\right)}(t,x)-\frac{1}{c^{2}t}\underset{\partial B_{\mathrm{ct}}(x)}{\int }(\bar{j}(0,y)-j_{s}(y))\;ds(y)\\ \;-\underset{B_{\mathrm{ct}}(0)}{\int }\frac{1}{\left|y\right|}\left({\mathrm{grad}}_{x}\bar{\rho }\left(t-\frac{\left|y\right|}{c},x+y\right)-\mathrm{grad}\rho_{s}(x+y)+\frac{1}{c^{2}}\partial_{t}\bar{j}\left(t-\frac{\left|y\right|}{c},x+y\right)\right)\;dy. \end{array}$$

Using that $\bar{j}(t,x)=j_{s}(x)$ for *t*≤0 and $\bar{\rho }(t,x)=\rho_{s}(x)-\overset{t}{\underset{-\infty }{\int }}{\text{div}}_{x}\bar{j}(\tau ,x)\;d\tau$, we find that

$$E(t,x)-E_{s}(x)=E^{\left(0\right)}(t,x)+\underset{R^{3}}{\int }\overset{t-\frac{\left|y\right|}{c}}{\underset{-\infty }{\int }}\frac{1}{\left|y\right|}{\mathrm{grad}}_{x}{\text{div}}_{x}\bar{j}(\tau ,x+y)\;d\tau \;dy-\frac{1}{c^{2}}\underset{R^{3}}{\int }\frac{1}{\left|y\right|}\partial_{t}\bar{j}\left(t-\frac{\left|y\right|}{c},x+y\right)\;dy.$$

□

### 3.4 Measurements

To simplify the calculations, we assume that the medium satisfies ***E***_s_=0 and ***B***_s_=0 in a domain Ω.

Next, we want to model the measurements of optical coherence tomography. We consider illuminating waves of the form

(30) $$E^{\left(0\right)}(t,x)=f\left(t+\frac{x_{3}}{c}\right)\eta$$

with a function $f\in C_{c}^{\infty }(R)$ and a fixed polarisation vector $\eta \in R^{2}\times \{0\}$, where we choose *f* such that ***E*** ^(0)^ fulfils the condition (24).

Moreover, we place point detectors everywhere on the detector surface

(31) $$D=R^{2}\times \{d\}$$

with *d* > 0 sufficiently large, in particular, we require that ***E*** ^(0)^(*t*,***ξ***) = 0 for *t*≥0 and ξ∈D. For more details, we refer to 11, Eqn ((29)].

Then, the reference wave ***E*** ^*z*^ produced by reflecting this incoming wave at a perfect mirror placed in the plane given by the equation *x*_3_=*z* is given by the following:

(32) $$E^{z}(t,x)=\left\{\begin{array}{ll} \left(f\left(t+\frac{x_{3}}{c}\right)-f\left(t+\frac{x_{3}}{c}+2\;\frac{z-x_{3}}{c}\right)\right)\eta & \text{if}\;x_{3}>z,\\ 0 & \text{if}\;x_{3}\leq z. \end{array}\right.$$

The measurements carried out in optical coherence tomography are now the intensities

(33) $$I_{j}(z,\xi )=\overset{\infty }{\underset{0}{\int }}|E_{j}(t,\xi )+E_{j}^{z}(t,\xi ){|}^{2}\;dt,\;\xi \in D,\;j\in \{1,2,3\},$$

on the detector surface D.

We combine these measurements to

(34) $${Ĩ}_{j}(z,\xi )=\frac{1}{2}\left(I_{j}(z,\xi )-\overset{\infty }{\underset{0}{\int }}{\left|E_{j}(t,\xi )\right|}^{2}\;dt-\overset{\infty }{\underset{0}{\int }}{\left|E_{j}^{z}(t,\xi )\right|}^{2}\;dt\right),$$

where the second term can be obtained by measuring the back‐scattered wave without superimposing the reflected wave ***E*** ^*z*^ and the last term is explicitly known from the initial laser pulse ***E*** ^(0)^. Equation (34) results to

$${Ĩ}_{j}(z,\xi )=\overset{\infty }{\underset{0}{\int }}E_{j}(t,\xi )E_{j}^{z}(t,\xi )\;dt.$$

Because we have by our choice of measurement setup that ***E***(*t*,***x***) = ***E*** ^(0)^(*t*,***x***) for *t*≤0 and ***E*** ^(0)^(*t*,***ξ***) = 0 for *t* > 0 and ξ∈D, we can write this in the form

$$\begin{array}{cc} {Ĩ}_{j}(z,\xi ) & =\overset{\infty }{\underset{0}{\int }}\left(E_{j}-E_{j}^{\left(0\right)}\right)(t,\xi )\left(E_{j}^{z}-E_{j}^{\left(0\right)}\right)(t,\xi )\;dt\\ =\overset{\infty }{\underset{-\infty }{\int }}\left(E_{j}-E_{j}^{\left(0\right)}\right)(t,\xi )\left(E_{j}^{z}-E_{j}^{\left(0\right)}\right)(t,\xi )\;dt. \end{array}$$

Inserting the explicit formula (32) for the reflected wave ***E*** ^*z*^, we obtain

(35) $${Ĩ}_{j}(z,\xi )=-\eta_{j}\overset{\infty }{\underset{-\infty }{\int }}\left(E_{j}-E_{j}^{\left(0\right)}\right)(t,\xi )f\left(t+\frac{2z-\xi_{3}}{c}\right)\;dt.$$

We use the convention

(36) $$\mathrm{FF}(\omega )=\overset{\infty }{\underset{-\infty }{\int }}F(t)e^{i\mathrm{\omega t}}\;dt$$

for the Fourier transform of an integrable function $F\in L^{1}(R)$ with respect to time, and we put a subindex at F if we need to specify the variable with respect to which we do the Fourier transform.

Taking the inverse Fourier transform with respect to *z* in (35) and using Plancherel's formula we obtain

$$\frac{2}{c}\overset{\infty }{\underset{-\infty }{\int }}{Ĩ}_{j}(z,\xi )e^{-i\frac{\omega }{c}(2z-\xi_{3})}\;dz=-\eta_{j}\left(F_{t}E_{j}-F_{t}E_{j}^{\left(0\right)}\right)(\omega ,\xi )\mathrm{Ff}(-\omega ).$$

Provided that the Fourier transform of the function *f* is nowhere zero and that the polarisation ***η*** fulfils *η*_1_≠0 and *η*_2_≠0, we obtain from this the data

$$h(t,\xi )=\left(\begin{array}{l} E_{1}(t,\xi )\\ E_{2}(t,\xi ) \end{array}\right),$$

where ***h*** can be directly calculated from the measurements ${Ĩ}_{j}$ and the knowledge of the initial wave ***E*** ^(0)^:

(37) $$F_{t}h_{j}(\omega ,\xi )=F_{t}E_{j}^{\left(0\right)}(\omega ,\xi )-\frac{2}{c\eta_{j}\mathrm{Ff}(-\omega )}\overset{\infty }{\underset{-\infty }{\int }}{Ĩ}_{j}(z,\xi )e^{-i\frac{\omega }{c}(2z-\xi_{3})}\;dz.$$

The inverse problem of optical coherence tomography is now to find the material parameter $\mu :R\times R^{3}\to R$ from the function $h:R\times D\to R^{2}$ according to the system of equations

(38) $$\begin{array}{ccc} \frac{1}{c^{2}}\partial_{\mathrm{tt}}E(t,x)+{\text{curl}}_{x}\;{\text{curl}}_{x}E(t,x) & =-\frac{4\pi }{c^{2}}\overset{\infty }{\underset{-\infty }{\int }}\partial_{t}\mu (\tau ,x)E(t-\tau ,x)\;d\tau ,\; & t>0,\;x\in R^{3},\\ E(t,x) & =f(t+\frac{x_{3}}{c})\eta \; & t\leq 0,\;x\in R^{3},\\ h(t,\xi ) & =\left(\begin{array}{l} E_{1}(t,\xi )\\ E_{2}(t,\xi ) \end{array}\right),\; & t>0,\;\xi \in D. \end{array}$$

At first, it seems that we have enough data to solve this problem: we have the three‐dimensional function ***h*** for every choice of function *f* and polarisation ***η***. However, because the problem is linear in the electric field ***E***, we do not gain any information by changing *f* or having more than two linearly independent polarisations ***η***. To see this, we perform a Fourier transform F with respect to time.

Lemma 3.6 Let $\mu :R\times R^{3}\to R$ be given, $\eta^{\left(k\right)}\in S^{1}\times \{0\}\subset R^{2}\times \{0\}$, *k* = 1,2,3, be so that the first two are linearly independent, and let *f* ^(*k*)^ be sufficiently regular functions so that the first two functions *f* ^(*k*)^ have a non‐vanishing Fourier transform:

$$Ff^{\left(k\right)}(\omega )\neq 0\;\text{for every}\;\omega \in R,\;k\in \{1,2\}.$$

Moreover, let ***E*** ^(*k*)^, *k* = 1,2,3, be the solutions of

$$\begin{array}{ccc} \frac{1}{c^{2}}\partial_{\mathrm{tt}}E^{\left(k\right)}(t,x)+{\text{curl}}_{x}\;{\text{curl}}_{x}E^{\left(k\right)}(t,x) & =-\frac{4\pi }{c^{2}}\overset{\infty }{\underset{-\infty }{\int }}\partial_{t}\mu (\tau ,x)E^{\left(k\right)}(t-\tau ,x)\;d\tau , & t>0,\;x\in R^{3},\\ E^{\left(k\right)}(t,x) & =f^{\left(k\right)}\left(t+\frac{x_{3}}{c}\right)\eta^{\left(k\right)}, & t\leq 0,\;x\in R^{3}. \end{array}$$

Then,the measurement data

(39) $$h^{\left(k\right)}(t,\xi )=\left(\begin{array}{l} E_{1}^{\left(k\right)}(t,\xi )\\ E_{2}^{\left(k\right)}(t,\xi ) \end{array}\right),\;t>0,\;\xi \in D,$$

fulfil the relation

(40) $$F_{t}h^{\left(3\right)}(\omega ,\xi )=\overset{2}{\underset{k=1}{\sum }}c_{k}\frac{Ff^{\left(3\right)}(\omega )}{Ff^{\left(k\right)}(\omega )}F_{t}h^{\left(k\right)}(\omega ,\xi ),\;\omega \in R,\;\xi \in D,$$

where the coefficients $c_{k}\in R$ are determined by $\eta^{\left(3\right)}=\overset{2}{\underset{k=1}{\sum }}c_{k}\eta^{\left(k\right)}$.

As in Proposition 3.5, we consider the equivalent integral equation (29) for ***E***_*s*_=0

$$\begin{array}{c} E^{\left(k\right)}(t,x)=f^{\left(k\right)}\left(t+\frac{x_{3}}{c}\right)\eta^{\left(k\right)}-\underset{R^{3}}{\int }\overset{t-\frac{\left|y\right|}{c}}{\underset{-\infty }{\int }}\frac{1}{\left|y\right|}\left({\mathrm{grad}}_{x}{\text{div}}_{x}-\frac{1}{c^{2}}\partial_{\mathrm{tt}}\right){\bar{j}}^{\left(k\right)}(\tau ,x+y)\;d\tau \;dy. \end{array}$$

Applying a Fourier transform with respect to time to the previous equation and using (20) in the frequency domain, we obtain that

(41) $$\begin{array}{cc} F_{t}E^{\left(k\right)}(\omega ,x) & =Ff^{\left(k\right)}(\omega )e^{-i\frac{\omega }{c}x_{3}}\eta^{\left(k\right)}+\left({\mathrm{grad}}_{x}{\text{div}}_{x}+\frac{\omega^{2}}{c^{2}}\right)\underset{R^{3}}{\int }\frac{ie^{i\frac{\omega }{c}|x-y|}}{\omega |x-y|}F_{t}\mu (\omega ,y)F_{t}E^{\left(k\right)}(\omega ,y)\;dy. \end{array}$$

Now, the linearity of this equation implies that the solution $F_{t}E^{\left(3\right)}$ of the third equation can be written in the form

$$F_{t}E^{\left(3\right)}(\omega ,x)=\overset{2}{\underset{k=1}{\sum }}c_{k}\frac{Ff^{\left(3\right)}(\omega )}{Ff^{\left(k\right)}(\omega )}F_{t}E^{\left(k\right)}(\omega ,x).$$

Restricting this relation to the detector surface D, we obtain (40). □

In particular, even if one considers the assumptions of Born and far‐field approximations (specified later), we see that the measurements are equivalent to the determination of the four‐dimensional Fourier transform of the function *μ* on a cone in $R^{3}$; see 11, Proposition 5.2]. Thus, also for OCT, we do not have sufficient data to uniquely recover the material parameter *μ*.

However, under certain simplifications, there exist works that provide reconstructions. A main assumption is that the medium is non‐dispersive, meaning that the temporal Fourier transform of *μ* does not depend on frequency. Under this assumption, Mark *et al.*, 41 proposed algorithms under the Born approximation, and in 42, 43, the one‐dimensional case was considered. Also in 9, 44, the OCT system is described using a single backscattering model.

## 4 Combined system

We have seen that the inverse problems of quantitative photoacoustic imaging and OCT both lack data to obtain a unique reconstruction of the material parameters depending on the specific modelling. Because the illumination for photoacoustic imaging and OCT is modelled analogously, they rely on the same electromagnetic material parameters of the medium. Therefore, we are suggesting to use the two measurements in combination to obtain additional informations.

To this end, we want to rewrite the optical problem in photoacoustics in terms of the material parameter *μ* (20) used in Maxwell's equations for the modelling of OCT instead of the absorption and scattering coefficients appearing in the radiative transfer equation. Thus, we need an expression of the absorbed energy of an electromagnetic wave in an dielectric medium.

We summarise the variables, which are used throughout this section, as a backup reference in Table 4.

**Table 4:** Combined PAT‐OCT system.

Symbol	Quantity	Definition
***η***	incident polarisation vector	(30)
***E*** ^(0)^	incident field	(30)
D	detector's array in OCT	(31)
***E*** ^*z*^	reflected field in OCT	(32)
*I*_*j*_	measured intensity in OCT	(33)
${Ĩ}_{j}$	effective measured intensity in OCT	(34)
***h***	OCT measurements	(37)
*p* ^(0)^	PAT measurements	(51c)
$\tilde{h}$	modified OCT measurements	(54)
$\tilde{p}$	modified PAT measurements	(57)

OCT, optical coherence tomography; PAT, photoacoustic tomography.

### 4.1 Absorbed energy

We therefore start again with Maxwell's microscopic equations (11), (13) and (14) to describe the medium and define the averaged mechanical energy as the sum of the kinetic energies of the particles and their electrostatic potential energy:

(42) $$E(t,x)=\overset{N}{\underset{i=1}{\sum }}\frac{m_{i}}{2}|x_{i}^{\prime }(t){|}^{2}w(x_{i}(t)-x)+\frac{1}{2}\underset{i\neq j}{\sum }\frac{q_{i}q_{j}}{\left|x_{i}(t)-x_{j}(t)\right|}w(x_{i}(t)-x)w(x_{j}(t)-x)\;.$$

Assuming now that no particles enter the support of *w*(·−***x***) or leave the region where *w* is constant, the change in energy is given by the following:

(43) $$\begin{array}{cc} \partial_{t}E(t,x) & =\overset{N}{\underset{i=1}{\sum }}m_{i}<x_{i}^{\prime }(t),x_{i}^{\prime \prime }(t)>w(x_{i}(t)-x)-\frac{1}{2}\underset{i\neq j}{\sum }q_{i}q_{j}\frac{<x_{i}(t)-x_{j}(t),x_{i}^{\prime }(t)-x_{j}^{\prime }(t)>}{|x_{i}(t)-x_{j}(t){|}^{3}}w(x_{i}(t)-x)w(x_{j}(t)-x)\\ =\overset{N}{\underset{i=1}{\sum }}m_{i}<x_{i}^{\prime }(t),x_{i}^{\prime \prime }(t)>w(x_{i}(t)-x)-\underset{i\neq j}{\sum }q_{i}q_{j}\frac{<x_{i}(t)-x_{j}(t),x_{i}^{\prime }(t)>}{|x_{i}(t)-x_{j}(t){|}^{3}}w(x_{i}(t)-x)w(x_{j}(t)-x). \end{array}$$

We consider the limit when the charge distributions *ρ*_*i*_ of the single particles tend to *δ*‐distributions, then the Lorentz force (14) is given by the following:

$$m_{i}x_{i}^{\prime \prime }(t)=q_{i}e(t,x_{i}(t))+q_{i}\frac{x_{i}^{\prime }(t)}{c}\times b(t,x_{i}(t)).$$

Using the identity (43), and the fact that $\frac{x_{i}^{\prime }}{c}\times b$ is orthogonal to $x_{i}^{\prime }$, we find that

$$\partial_{t}E(t,x)=\overset{N}{\underset{i=1}{\sum }}q_{i}<x_{i}^{\prime }(t),e(t,x_{i}(t))>w(x_{i}(t)-x)-\underset{i\neq j}{\sum }q_{i}q_{j}\frac{<x_{i}(t)-x_{j}(t),x_{i}^{\prime }(t)>}{|x_{i}(t)-x_{j}(t){|}^{3}}w(x_{i}(t)-x)w(x_{j}(t)-x).$$

We further assume that the particles are moving relatively slow so that we may approximate the electric field ***e*** by the electrostatic approximation, which can be represented via (A2) by neglecting the contribution of the current ***j***:

$$\begin{array}{cc} e(t,x) & \approx e^{\left(0\right)}(t,x)-\underset{B_{\mathrm{ct}}(0)}{\int }\frac{1}{\left|y\right|}{\mathrm{grad}}_{x}\rho (t,x+y)\;dy\\ \approx e^{\left(0\right)}(t,x)-\overset{N}{\underset{j=1}{\sum }}q_{j}\underset{R^{3}}{\int }\frac{1}{\left|y\right|}{\mathrm{grad}}_{x}\delta (x+y-x_{j}(t))\;dy\\ \approx e^{\left(0\right)}(t,x)+\overset{N}{\underset{j=1}{\sum }}q_{j}\frac{x-x_{j}(t)}{|x-x_{j}(t){|}^{3}}. \end{array}$$

where ***e*** ^(0)^ denotes the solution of the homogeneous wave equation with initial data ***e*** ^(0)^(0,***x***) = ***e***(0,***x***) and *∂*_*t*_ ***e*** ^(0)^(0,***x***) = **curl**_***x***_ ***b***(0,***x***). Here, we have used that for *t* sufficiently large, *B*_*ct*_(0) will contain the complete medium and (13a) where *ρ*_*i*_ is replaced by a *δ*‐distribution.

Then, we can write the change in energy in the form

(44) $$\partial_{t}E(t,x)\approx \overset{N}{\underset{i=1}{\sum }}q_{i}<x_{i}^{\prime }(t),e^{\left(0\right)}(t,x_{i}(t))>w(x_{i}(t)-x)+\underset{i\neq j}{\sum }q_{i}q_{j}\frac{<x_{i}(t)-x_{j}(t),x_{i}^{\prime }(t)>}{|x_{i}(t)-x_{j}(t){|}^{3}}w(x_{i}(t)-x)(1-w(x_{j}(t)-x)).$$

Until now, we had no particular assumptions on the size of the support of *w*. We have seen that the particles have diameter of order 10^−14^ m. The size of molecules in biological tissue is of order 10^−10^ m; a water molecule for example has a diameter of 0.3 nm. Thus, we specify *r* to be of order 10^−10^ m. In what follows, we assume in addition to $w\in C_{c}^{\infty }(R^{3})$ that

(45) $$w\equiv \text{const in}B_{r}(0)\text{and}w\equiv 0\text{outside}B_{r+\epsilon }(0)\;.$$

Practically, the typical wavelength of photoacoustic imaging and OCT is around 1000 nm, which is three orders of magnitude larger than the size of molecules. Then, a ball with radius $\tilde{r}$ of order 10^−6^ m will denote the resolution limitation, and we can assume that $\tilde{r}\gg r$.

For the averaging here, we assume that ***e*** ^(0)^ is almost constant on the support of *w*(·−***x***). Then, we may approximate

(46) $$e^{\left(0\right)}(t,x_{i}(t))\approx \underset{R^{3}}{\int }e^{\left(0\right)}(t,y)w(y-x)\;dy,\;x_{i}(t)\in B_{r}(x)\;.$$

The second term in (44) is nonzero only when ***x***_*i*_∈*B*_*r*_(***x***) and ***x***_*j*_∉*B*_*r*_(***x***). We want to assume that locally (related to resolution limit), there is almost no energy exchange:

$$\underset{i\neq j}{\sum }q_{i}q_{j}\frac{<x_{i}(t)-x_{j}(t),x_{i}^{\prime }(t)>}{|x_{i}(t)-x_{j}(t){|}^{3}}w(x_{i}(t)-x)(1-w(x_{j}(t)-x))\approx 0,\;x_{j}(t)\in B_{\tilde{r}}(x)\setminus B_{r}(x).$$

For the case, where ***x***_*i*_∈*B*_*r*_(***x***) and $x_{j}\notin B_{\tilde{r}}(x)$, because $\tilde{r}\gg r$, we can approximate ***x***_*i*_−***x***_*j*_≃***y*** − ***x***_*j*_, for every ***y***∈*B*_*r*_(***x***), and we thus obtain

$$\begin{array}{cc} \partial_{t}E(t,x) & \approx <\underset{R^{3}}{\int }e^{\left(0\right)}(t,y)w(y-x)\;dy,\overset{N}{\underset{i=1}{\sum }}q_{i}x_{i}^{\prime }(t)w(x_{i}(t)-x)>\\ \;+<\overset{N}{\underset{j=1}{\sum }}q_{j}\underset{R^{3}}{\int }\frac{y-x_{j}(t)}{|y-x_{j}(t){|}^{3}}w(y-x)\;dy(1-w(x_{j}(t)-x)),\overset{N}{\underset{i=1}{\sum }}q_{i}x_{i}^{\prime }(t)w(x_{i}(t)-x)>. \end{array}$$

Now, because the support of *w* is small compared with the medium, we may take in the second term the sum over all particles ***x***_*j*_ without introducing a large error and find that

$$\partial_{t}E(t,x)\approx <E(t,x),\bar{j}(t,x)>.$$

Thus, in a non‐magnetic, isotropic and linear dielectric medium, we have that the total absorbed energy is given by the following:

(47) $$\underset{t\to \infty }{\lim}E(t,x)-\underset{t\to -\infty }{\lim}E(t,x)\approx \overset{\infty }{\underset{-\infty }{\int }}\overset{\infty }{\underset{-\infty }{\int }}\mu (\tau ,x)<E(t,x),E(t-\tau ,x)>\;d\tau \;dt.$$

Remark 4.1 A different derivation of the absorbed energy follows from the definition of the total electromagnetic energy density and the energy transport of the electromagnetic wave (Poynting vector) as follows:

(48) $$W=\frac{1}{8\pi }(〈E,D〉+〈B,B〉),\;\text{and}\;S=\frac{c}{4\pi }(E\times B),$$

respectively. Here,

(49) D=E+4πP

denotes the electric displacement. Using the elementary relation div_***x***_(***F*** × ***G***) = ***G***·**curl**_***x***_ ***F*** − ***F***·**curl**_***x***_ ***G***, the Maxwell's equations (15c) and (15d) follow that

$${\text{div}}_{x}S=-\frac{1}{4\pi }\left(〈B,\partial_{t}B〉+〈E,\partial_{t}E〉\right)-〈E,\bar{j}〉.$$

Then, from the definition of W, integrating the previous equation over a subvolume Ω of the medium and applying the divergence theorem give

(50) $$-\partial_{t}\underset{\Omega }{\int }W(t,x)\;dx=\underset{\partial \Omega }{\int }〈S(t,x),N〉\;ds(x)+\underset{\Omega }{\int }〈E(t,x),\bar{j}(t,x)〉\;dx-\frac{1}{2}\underset{\Omega }{\int }\partial_{t}〈E(t,x),P(t,x)〉\;dx$$

where ***N*** is the outward pointing unit normal vector.

Eqn (50) describes the balance of energy: by the definition of W, the left hand side quantifies the variation of the total energy in the volume. The first term on the right‐hand side describes the variation of the energy across the boundary, and the last two terms describe the dissipated power. According to 40, the integrands of the last two terms in the right‐hand side integrated over time denote the electric energy density, which reads as follows:

$$\begin{array}{cc} \overset{\infty }{\underset{-\infty }{\int }}〈E(t,x),\bar{j}(t,x)〉\;dt & =\overset{\infty }{\underset{-\infty }{\int }}\overset{\infty }{\underset{-\infty }{\int }}\mu (\tau ,x)〈E(t,x),E(t-\tau ,x)〉\;d\tau \;dt, \end{array}$$

resulting again in (47), if we assume that the polarisation goes to zero after a long time.

### 4.2 Inverse problem

Using the expression (47) for the absorbed energy as a replacement of $\mu_{a}(x)\bar{\Phi }(x)$ in (6), we obtain by combining (6) and (38) the inverse problem of calculating *μ* and *γ* from the measurement data *p* ^(0)^ and *h* for giving initial pulse *f* and polarisation ***η*** obeying the equation system

$$\begin{array}{ccc} \frac{1}{c^{2}}\partial_{\mathrm{tt}}E(t,x) & =-{\text{curl}}_{x}\;{\text{curl}}_{x}E(t,x)-\frac{4\pi }{c^{2}}\overset{\infty }{\underset{-\infty }{\int }}\partial_{t}\mu (\tau ,x)E(t-\tau ,x)\;d\tau ,\; & t>0,\;x\in R^{3},\\ E(t,x) & =f(t+\frac{x_{3}}{c})\eta ,\; & t\leq 0,\;x\in R^{3},\\ p^{\left(0\right)}(x) & =\gamma (x)\overset{\infty }{\underset{-\infty }{\int }}\overset{\infty }{\underset{-\infty }{\int }}\mu (\tau ,x)<E(t,x),E(t-\tau ,x)>\;d\tau \;dt,\;\; & \;\;\;\;\;x\in R^{3},\\ h(t,\xi ) & =\left(\begin{array}{l} E_{1}(t,\xi )\\ E_{2}(t,\xi ) \end{array}\right),\; & t>0,\;\xi \in D. \end{array}$$

Now, although varying the initial pulse *f* does not give us additional information with respect to the measurements of OCT, Lemma 3.6, its influence in the photoacoustic part is nonlinear and provides us with independent data. Thus, by choosing a one‐parameter family of functions *f*, for example, almost monochromatic waves *f*_*ν*_ centred at a frequency *ν* for arbitrary *ν* > 0, we obtain for every *ν* > 0 the equation system

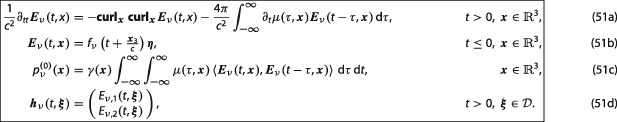

Now, the first two equations uniquely determine ***E***_*ν*_ as a function of *μ*. Then, depending on *γ*, we would have to solve the third equation for the four‐dimensional function *μ*, which would leave us with two three‐dimensional equations for the three‐dimensional Grüneisen parameter *γ*.

Although the dimensions of the data seem to perfectly fit for a reconstruction, it is still an open problem if these equations uniquely determine the material parameters; although there are partial results in this direction, see 17, where the injectivity of a very similar system is discussed.

As a plausibility argument, we want to show in the next section that at least in a simplified case, the parameters can be uniquely recovered.

## 5 Weakly scattering medium and multiple pulsed laser illuminations

The full system (51) can be equivalently transformed to a Fredholm integral equation of the second kind under the following assumptions:

- Born approximation: the medium is weakly scattering, meaning that $F_{t}\mu$ is sufficiently small.
- Far‐field approximation: typically, in an OCT system, the measurements are performed in a distance much bigger compared with the size of the medium.
- Specific illumination: the support of the initial pulses tends to a delta distribution such that the optical parameter can be assumed constant in this spectrum.

In a first step, we derive a Fredholm integral equation of the first kind for the unknown Grüneisen parameter.

Proposition 5.1 The complete system (51) under the previous three assumptions is reduced to the integral equation

(52) $$\underset{R^{3}}{\int }K[\tilde{p}](\nu ,\vartheta ;y)\frac{1}{\gamma (y)}\;dy=\tilde{h}(\nu ,\vartheta )$$

where

(53) $$K[\tilde{p}](\nu ,\vartheta ;y)=\left(\tilde{p}(\nu ,y)-\frac{i}{\pi }\overset{\infty }{\underset{-\infty }{\int }}\frac{\tilde{p}(\tilde{\nu },y)}{\tilde{\nu }-\nu }\;d\tilde{\nu }\right)e^{-i\frac{\nu }{c}<\vartheta +e_{3},y>},\;\tilde{p}(\nu ,x):=2\pi p_{\nu }^{\left(0\right)}(x)$$

and

(54) $$\tilde{h}(\nu ,\vartheta )=\overset{2}{\underset{j=1}{\sum }}\left(\frac{F_{t}E_{\nu }^{\left(b\right)}(\nu ,R\vartheta )}{Ff_{\nu }(\nu )}-e^{-i\frac{\nu }{c}R\vartheta_{3}}\eta_{j}\right)\frac{\mathrm{iR}c^{2}e^{-i\frac{\nu }{c}R}}{\nu {\left(\vartheta \times \vartheta \times \eta \right)}_{j}}.$$

We start with (51a) and (51b). We recall Eqn (41) derived in the proof of Lemma 3.6, then we obtain

$$\begin{array}{cc} F_{t}E_{\nu }(\omega ,x) & =Ff_{\nu }(\omega )e^{-i\frac{\omega }{c}x_{3}}\eta +\left({\mathrm{grad}}_{x}{\text{div}}_{x}+\frac{\omega^{2}}{c^{2}}\right)\underset{R^{3}}{\int }\frac{ie^{i\frac{\omega }{c}|x-y|}}{\omega |x-y|}F_{t}\mu (\omega ,y)F_{t}E_{\nu }(\omega ,y)\;dy \end{array}$$

that holds for every frequency *ν* > 0. Under the Born approximation, we replace $F_{t}E_{\nu }$ in the integral by the incident field, to obtain $E_{\nu }^{\left(b\right)}$:

$$\begin{array}{c} F_{t}E_{\nu }^{\left(b\right)}(\omega ,x)=Ff_{\nu }(\omega )\left(e^{-i\frac{\omega }{c}x_{3}}\eta +\left({\mathrm{grad}}_{x}{\text{div}}_{x}+\frac{\omega^{2}}{c^{2}}\right)\underset{R^{3}}{\int }\frac{ie^{i\frac{\omega }{c}(|x-y|-y_{3})}}{\omega |x-y|}F_{t}\mu (\omega ,y)\;dy\;\eta \right). \end{array}$$

We set ***x*** = *R* ***ϑ***,*R* > 0 and $\vartheta \in S^{2}$. Considering now the far‐field approximation, we can replace the previous expression by its asymptotic behaviour for *R*→*∞*, uniformly in ***ϑ***. This gives us, see for example 11, Equation (4.1) in Chapter 4.1]:

(55) $$F_{t}E_{\nu }^{\left(b\right)}(\omega ,\mathrm{R\vartheta})≃Ff_{\nu }(\omega )\left(e^{-i\frac{\omega }{c}R\vartheta_{3}}\eta -\frac{i\omega e^{i\frac{\omega }{c}R}}{Rc^{2}}\vartheta \times \vartheta \times \eta \underset{R^{3}}{\int }e^{-i\frac{\omega }{c}<\vartheta +e_{3},y>}F_{t}\mu (\omega ,y)\;dy\right).$$

Then, for every $\vartheta \in \tilde{D}=\{\theta \in S^{2}|\exists R>0:R\theta \in D\}$ and $\nu \in R^{+}$, the previous equation using Eqn (51d) and the definition (54) can be rewritten as follows:

(56) $$\tilde{h}(\nu ,\vartheta )=\underset{R^{3}}{\int }e^{-i\frac{\nu }{c}<\vartheta +e_{3},y>}F_{t}\mu (\nu ,y)\;dy,$$

where we assumed that $Ff_{\nu }(\nu )\neq 0$.

Similarly, we proceed with the Eqn (51c) of the photoacoustic tomography (PAT) measurement, written as follows:

$$p_{\nu }^{\left(0\right)}(x)=\gamma (x)\overset{\infty }{\underset{-\infty }{\int }}<E_{\nu }(t,x),\overset{\infty }{\underset{-\infty }{\int }}\mu (\tau ,x)E_{\nu }(t-\tau ,x)\;d\tau >\;dt.$$

From Plancherel's formula, it follows that

$$\begin{array}{cc} p_{\nu }^{\left(0\right)}(x) & =\gamma (x)\frac{1}{2\pi }\overset{\infty }{\underset{-\infty }{\int }}<F_{t}E_{\nu }(\omega ,x),\bar{F_{t}\mu (\omega ,x)F_{t}E_{\nu }(\omega ,x)}>\;d\omega \\ =\gamma (x)\frac{1}{2\pi }\overset{\infty }{\underset{-\infty }{\int }}\bar{F_{t}\mu (\omega ,x)}|F_{t}E_{\nu }(\omega ,x){|}^{2}\;d\omega \\ =\gamma (x)\frac{1}{2\pi }\overset{\infty }{\underset{-\infty }{\int }}F_{t}\mu (\omega ,x)|F_{t}E_{\nu }(\omega ,x){|}^{2}\;d\omega . \end{array}$$

For the last equation, we used that $p_{\nu }^{\left(0\right)}$ is real valued. Considering again the Born approximation, we obtain

$$\tilde{p}(\nu ,x)=\gamma (x)\overset{\infty }{\underset{-\infty }{\int }}F_{t}\mu (\omega ,x)|Ff_{\nu }(\omega ){|}^{2}\;d\omega .$$

To take advantage of the third assumption, we choose initial pulses so that $Ff_{\nu }$ has only support on a domain {ω∈R∣|ω|∈[ν−ϵ,ν+ϵ]} with a sufficiently small *ϵ* > 0 such that $F_{t}\mu (\cdot ,x)$ can be assumed to be for every $x\in R^{3}$ constant on this support (we remark that because *f* and *μ* are a real‐valued functions, the real and imaginary parts of their Fourier transforms have to be even and odd, respectively). If we additionally normalise $\overset{\infty }{\underset{-\infty }{\int }}|Ff_{\nu }(\omega ){|}^{2}\;d\omega =\frac{1}{2}$, we find that

(57) $$\tilde{p}(\nu ,x)=\gamma (x)Re(F_{t}\mu (\nu ,x)).$$

Moreover, because *μ* is a real‐valued function, we know that its Fourier transform fulfils the Kramers–Kronig relation 45

(58) $$I(F_{t}\mu (\omega ,x))=-\frac{1}{\pi }\overset{\infty }{\underset{-\infty }{\int }}\frac{Re(F_{t}\mu (\tilde{\omega },x))}{\tilde{\omega }-\omega }\;d\tilde{\omega }.$$

Thus, combining (56), (57) and (58), we obtain the integral equation (52) for the function $\frac{1}{\gamma }$ where the kernel depends on the data $\tilde{p}$ obtained from the PAT measurements. □

Remark 5.2

- Compared with 11, Formula (20)], we have to mention that the different term in front of the integral in Eqn (55) is due to the different scaling between the coefficients *μ* and *χ*; see the definition of ***P***.
- In practice, we do not have Eqn (56) for every $\nu \in R^{+}$ because the band‐limited source allows measurements only for a fixed frequency spectrum.
- In the most commonly used formulas, the imaginary part of the permittivity or the conductivity appears in (57). The differentiation of our result is due to the definition of *μ*, Remark 3.4, and should not confuse the reader.

### 5.1 Analytical formulas for special material

The particular form of the kernel *K*, Eqn (53), motivated us to transform the integral equation (52) of the first kind to one of the second kind. More precisely, in the special case, where the object mainly consists of a single material with the frequency dependence given by some function *α* and a density distribution *β*, that is, if the initial pressure is of the form

$$\tilde{p}(\nu ,x)=\alpha (\nu )\beta (x)+\epsilon (\nu ,x)$$

for some small function *ϵ*, we may write the integral equation (52) considering (53) as follows:

$$A(\nu )\underset{R^{3}}{\int }\frac{\beta (y)}{\gamma (y)}e^{-i\frac{\nu }{c}<\vartheta +e_{3},y>}\;dy+\underset{R^{3}}{\int }K[\epsilon ](\nu ,\vartheta ;y)\frac{\beta (y)}{\gamma (y)}\;dy=\tilde{h}(\nu ,\vartheta ),$$

where

$$A(\nu )=\alpha (\nu )-\frac{i}{\pi }\overset{\infty }{\underset{-\infty }{\int }}\frac{\alpha (\tilde{\nu })}{\tilde{\nu }-\nu }\;d\tilde{\nu }.$$

If we assume that *A*(*ν*) ≠ 0, then we may rewrite this as a Fredholm integral equation of the second kind for the Fourier transform

$$\Gamma (k)=\underset{R^{3}}{\int }\frac{\beta (y)}{\gamma (y)}e^{-i<k,y>}\;dy$$

of the function $\frac{\beta }{\gamma }$:

$$\Gamma (k)+\frac{1}{A(\nu )}\underset{R^{3}}{\int }\hat{K}(k;\kappa )\Gamma (\kappa )\;d\kappa =ĥ(k),$$

where the kernel $\hat{K}$ is for all values $k\in R^{3}$, which are of the form $k=\frac{\nu }{c}(\vartheta +e_{3})$, $\vartheta \in \tilde{D}$, defined by the following:

$$\hat{K}\left(\frac{\nu }{c}(\vartheta +e_{3});\kappa \right)=\frac{1}{A(\nu )}\frac{1}{{\left(2\pi \right)}^{3}}\underset{R^{3}}{\int }K[\epsilon ](\nu ,\vartheta ;y)e^{i<\kappa ,y>}\;dy$$

and ĥ is similarly defined by the following:

$$ĥ\left(\frac{\nu }{c}(\vartheta +e_{3})\right)=\frac{1}{A(\nu )}\tilde{h}(\nu ,\vartheta ).$$

In particular, we see that for a sufficiently small function *ϵ*, the integral equation for Γ can have at most one solution 46, 47.

## 6 Relation between the material parameters

In this section, we want to discuss the connection between the parameter *μ*, defined in (20), and the absorption and scattering coefficients, *μ*_a_ and *μ*_s_, appearing in the radiative transfer equation (3). Recently, there has been an established connection between the radiative transfer equation (vector and scalar) and equations in classical electromagnetism 48, 49. However, even under simplifying assumptions, these results do not lead to a direct connection between the material parameters *μ*, *μ*_a_ and *μ*_s_ as the following remark shows.

Remark 6.1 This remark is based on classical results 50, 51 and follows 49. Let the medium contain *N* well separated scattering particles. We assume that there exist balls *B*_*k*_,*k* = 1,...,*N* containing only one particle with large radius. We define by ***N***_*k*_ the unit outward normal vector on the boundary *∂* *B*_*k*_. Let us denote by ***F*** ^+^ and ***F*** ^−^ the outgoing (scattered) and incoming (incident) field for a given particle, respectively. If we neglect the interference effects between the incoming and outgoing fields, we can set as incident on the subdomain *B*_*k*_ the field

$$E_{k}^{-}=\underset{x\in \Gamma_{k}}{\sum }E,\;\text{where}\;\Gamma_{k}=\left\{x\in \partial B_{k}|〈S,N_{k}〉<0\right\},$$

where ***S*** is defined in Remark 4.1. Then, $E_{k}^{+}=E-E_{k}^{-}$. We redefine the time averaged Poynting vector (the real part of the complex Poynting vector)

$$S^{\alpha }(\nu ,x):=\frac{c}{8\pi }Re\left(F_{t}E^{\alpha }(\nu ,x)\times \bar{F_{t}B^{\alpha }(\nu ,x)}\right),\;\alpha =-,+,$$

describing the average flux of the incident and the scattered field, respectively. Then, the averaged rate of the energy incident on *k*th particle is given by $S_{k}^{-}(\nu ):=\frac{1}{B_{k}}\underset{B_{k}}{\int }|S^{-}(\nu ,x)|\;dx$.

Then, because every domain contains only one particle, the coefficients obtain the forms

$$\begin{array}{cc} \mu_{a,k}(\nu ) & =\frac{B_{k}}{S_{k}^{-}(\nu )}\underset{B_{k}}{\int }\underset{R}{\int }〈E(t,x),\bar{j}(t,x)〉\;dt\;dx,\;k=1,...,N\\ \mu_{s,k}(\nu ) & =\frac{B_{k}}{S_{k}^{-}(\nu )}\underset{\partial B_{k}}{\int }〈S^{+}(\nu ,x),N_{k}〉\;ds(x),\;k=1,...,N. \end{array}$$

If the assumptions of the previous section still hold, then we can approximate the absorbed power as in the derivation of (57) resulting to

$$\mu_{a,k}(\nu )≃\frac{B_{k}}{S_{k}^{-}(\nu )}\underset{B_{k}}{\int }Re(F_{t}\mu (\nu ,x))\;dx.$$

Observing the previous relations, we can identify the connection between the real part of the Fourier transform of *μ* and the absorption coefficient. But still arises the question if the obtained absorption and scattering coefficients are the ones that appear in the RTE; see Section 2. The fact that they depend on the incident average rate results to an indirect dependence on the initial illumination characterising the coefficients as non material parameters. If we omit the Born approximation, the dependence on the electric fields is stronger. In addition, the assumptions on the inner structure of the medium make the previous formulas applicable only in special cases.

## 7 Conclusions

To our knowledge, this paper is the first to use Maxwell's equations to model PAT. So far, the Maxwell's equations were only considered for modelling thermoacoustic tomography where low‐frequency radiation is used. However, because recently, the connection between the radiative transfer equation and classical electromagnetism has been established 48, 49, there is no need to differentiate from the proposed model considering the different modalities photoacoustic or thermoacoustic. Additionally, in our model, the frequency dependence of the optical parameter is not neglected, and thus, the effect of the different incident frequencies to the medium is included. This modelling allows to develop a unifying model for OCT and PAT, and thus, we can describe the combined setup.

## Appendix A Solution of Maxwell's equations

### A.1

If the charge density *ϱ* and the electric current ***j*** are known, then Maxwell's microscopic equations (11) can be explicitly solved. Proposition A.1 Let ***e*** and ***b*** be solutions of (11) for given functions *ρ* and ***j***. Then, ***e*** has the form

(A1) $$\begin{array}{cc} e(t,x) & =\frac{1}{4\mathrm{\pi ct}}\underset{\partial B_{\mathrm{ct}}(x)}{\int }\left({\text{curl}}_{x}b(0,y)-\frac{4\pi }{c}j(0,y)\right)\;ds(y)+\partial_{t}\left(\frac{1}{4\pi c^{2}t}\underset{\partial B_{\mathrm{ct}}(x)}{\int }e(0,y)\;ds(y)\right)\\ \;-\underset{B_{\mathrm{ct}}(0)}{\int }\frac{1}{\left|y\right|}\left({\mathrm{grad}}_{x}\rho \left(t-\frac{\left|y\right|}{c},x+y\right)+\frac{1}{c^{2}}\partial_{t}j\left(t-\frac{\left|y\right|}{c},x+y\right)\right)\;dy. \end{array}$$

We combine the Eqns (11c) and (11d) to the vector wave equation

$$\frac{1}{c^{2}}\partial_{\mathrm{tt}}e(t,x)+{\text{curl}}_{x}\;{\text{curl}}_{x}e(t,x)=-\frac{4\pi }{c^{2}}\partial_{t}j(t,x).$$

Then, using the vector identity **curl**_***x***_ **curl**_***x***_ ***e***(*t*,***x***) = **g** **r** **a** **d**_***x***_div_***x***_ ***e***(*t*,***x***)−Δ_*x*_ ***e***(*t*,***x***), we can transform this with (11a) to the inhomogeneous wave equation

$$\frac{1}{c^{2}}\partial_{\mathrm{tt}}e(t,x)-\Delta_{x}e(t,x)=-4\pi f(t,x),\;f(t,x)={\mathrm{grad}}_{x}\rho (t,x)+\frac{1}{c^{2}}\partial_{t}j(t,x).$$

Subtracting the solution ***e*** ^(0)^ of the homogeneous wave equation with the initial conditions ***e*** ^(0)^(0,***x***) = ***e***(0,***x***) and $\partial_{t}e^{\left(0\right)}(0,x)={\text{curl}}_{x}b(0,x)-\frac{4\pi }{c}j(0,x)$, we find that ***e*** − ***e*** ^(0)^ solves the inhomogeneous wave equation with zero initial data. So, by Duhamel's principle, for example, 52, Chapter 2.4, Theorem 4], the solution is given by the following:

$$e(t,x)=e^{\left(0\right)}(t,x)-\overset{\mathrm{ct}}{\underset{0}{\int }}\frac{1}{\mathrm{ct}-\zeta }\underset{\partial B_{\mathrm{ct}-\zeta }(x)}{\int }f\left(\frac{\zeta }{c},y\right)\;ds(y)\;d\zeta .$$

Combining the two integrals, we obtain

(A2) $$e(t,x)=e^{\left(0\right)}(t,x)-\underset{B_{\mathrm{ct}}(x)}{\int }\frac{1}{\left|y-x\right|}f\left(t-\frac{\left|y-x\right|}{c},y\right)\;dy.$$

Plugging in the explicit formula for the solution ***e*** ^(0)^ of the homogeneous wave equation, as in, 52, Chapter 2.4, Theorem 2], we arrive at (A1). □
